# Intermittent Fasting and Physical Exercise for Preventing Metabolic Disorders through Interaction with Gut Microbiota: A Review

**DOI:** 10.3390/nu15102277

**Published:** 2023-05-11

**Authors:** Li Zhang, Yuanshang Wang, Ying Sun, Xin Zhang

**Affiliations:** 1 Department of Physical Education, China University of Mining and Technology, Beijing 100083, China; zhangli304036@126.com (L.Z.); 13188818181@163.com (Y.W.); 2 Department of Food Science and Engineering, Ningbo University, Ningbo 315211, China; sysy42365@163.com

**Keywords:** metabolic disorders, gut microbiota, intermittent fasting, physical exercise

## Abstract

Metabolic disorders entail both health risks and economic burdens to our society. A considerable part of the cause of metabolic disorders is mediated by the gut microbiota. The gut microbial structure and function are susceptible to dietary patterns and host physiological activities. A sedentary lifestyle accompanied by unhealthy eating habits propels the release of harmful metabolites, which impair the intestinal barrier, thereby triggering a constant change in the immune system and biochemical signals. Noteworthy, healthy dietary interventions, such as intermittent fasting, coupled with regular physical exercise can improve several metabolic and inflammatory parameters, resulting in stronger beneficial actions for metabolic health. In this review, the current progress on how gut microbiota may link to the mechanistic basis of common metabolic disorders was discussed. We also highlight the independent and synergistic effects of fasting and exercise interventions on metabolic health and provide perspectives for preventing metabolic disorders.

## 1. Introduction

Overnutrition and unhealthy eating habits, coupled with urbanization and sedentary occupations, dramatically boost the development of metabolic disorders. Metabolic disorders represent a series of complex conditions characterized by abdominal obesity, dyslipidaemia, hypertension, glucose intolerance, and insulin resistance that, when occurring together, strongly lead to detrimental clinical outcomes, entailing both health risks and economic burdens to our society. Current estimates report that the incidence rate of metabolic diseases is increasing year by year, with a projected cost in excess of 5000 USD for each individual. Up to 2019, it is estimated that there are 43.8 million cases of type 2 diabetes (T2D), 18.5 million cases of hypertension, and 1.2 billion cases of nonalcoholic fatty liver disease (NAFLD) in the world. Data on the incidence rates of obesity and hyperlipidemia have not been reported yet, but the mortality rates of both are the highest. In 2019 alone, 5 million people died of obesity and 4.3 million died of hyperlipidemia [1]. From the results of physiological and omics-based research, complemented by experiments in cells and animals, it appears that a considerable part of the cause of metabolic disorders is mediated by the gut microbiota [2,3].

The human body is contextualized by the coexisting microorganisms living in the digestive tract, called the gut microbiota. The gut microbiota is a crucial actor in digesting food, producing a diverse reservoir of metabolites to modify human metabolism, or triggering host reactions that mediate physiological processes [4]. The pioneering evidence regarding the mechanistic involvement of the gut microbiota in metabolic equilibrium was provided in 2004, suggesting that the capacity of the host for harvesting energy from the diet and energy storage was influenced by gut microbiota [5]. Subsequently, the research field has delivered a substantial amount of new knowledge about the potential effects of the gut microbiota in metabolic disorders, spearheaded by the application of next-generation microbiome sequencing and targeted bioinformatics [4]. The robustness and function of gut microbiota partly depend on the host, but are also modified by the environment (e.g., irregular smoking, excessive drinking, prolonged sleep deprivation, and a high-fat or high-sugar diet), particularly the dietary habits, which could act in concert to favor a healthy or diseased status [2]. In general, the ratio between Bacteroidetes and Firmicutes remains relatively stable in healthy individuals, which contributes to producing beneficial metabolites for health. Conversely, the microbiota of individuals with metabolic disorders is often characterized by the exasperation of the Firmicutes phylum and the reduction of the Bacteroidetes phylum, poor microbial diversity, decreased short-chain fat acid (SCFA) production, as well as enhanced intestinal permeability compared with that of healthy individuals [6].

Intermittent fasting (IF) is a dietary intervention pattern that targets a specific period of time (ranging from a few hours to several days) to stop dietary intake and consume a little or no calories, which has gained popularity in recent years and is expected to become a possible new paradigm in the avenue to improve body health [7]. Patterns of the specific IF diets include time-restricted feeding (TRF), alternate-day fasting (ADF), and the 5:2 strategy [2]. In TRF, a subset may consist of 16 h fasts with an 8 h nutritional window, 20 h fasts with a 4 h nutritional window, or other similar versions. ADF consists of a 24 h fasting period to consume 25% of the daily energy intake, alternated with a 24 h eating period that can be performed several times a week. Another 5:2 strategy is 2 fast days interspersed with 5 nonrestrictive days [8]. Importantly, the evolution of the metabolic, endocrine, and nervous systems in the fasted state can allow the optimization of physical and behavioral performance. A large number of cross-sectional and longitudinal studies have demonstrated the robust disease-modifying efficacy of the IF by interacting with the gut microbiota [7,8,9]. IF intervention results in a bloom of health-associated microbiota, contributing to the production of beneficial fermentation products, such as acetate and lactate, and dramatically ameliorating obesity, insulin resistance, and hepatic steatosis [10].

Physical exercise is another important way to prevent metabolic disorders. Physical exercise is explained as “a subset of physical activity that is planned, structured and repetitive”, and gears to either improve or maintain physical fitness [11]. Moderate physical exercise can help prevent excess weight gain by promoting a cascade of favorable changes in the metabolic homeostasis of the human body [12]. Healthy dietary patterns coupled with regular physical exercise improve several metabolic and inflammatory parameters in chronic diseases. In this review, we will discuss the potential impacts and mechanisms of gut microbiota in metabolic disorders and sum up the current research findings on the independent effects of IF and exercise in metabolic disorders. We also shed light on the benefits of combined exercise and IF interventions for metabolic disorders.

## 2. Mechanistic Insights of Gut Microbiota in Metabolic Disorders

**Figure 1:**
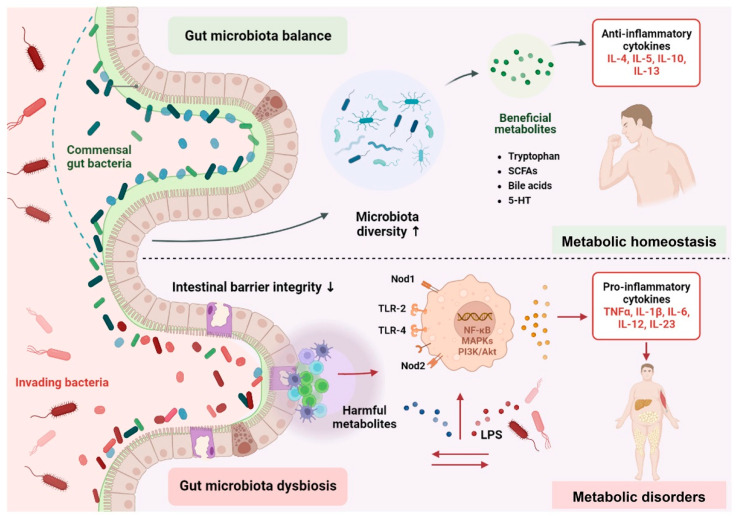
The underlying regulatory mechanisms of the gut microbiota in metabolic disorders. ↑ = up-regulate, ↓ = down-regulate.

Although the incidence of metabolic disorders is correlated with genetic and environmental factors, evidence is proliferating for the role of the gut microbiota in metabolic disorders. The underlying regulatory mechanisms are complex (Figure 1). The gut microbiota can produce beneficial metabolites, deriving directly from bacteria or the transformation of dietary or host-derived substrates, which contribute to maintaining metabolic health. Probiotics, such as *Pediococcus pentococcus* PP04 and *Lactobacillus plantarum* LP104, can also affect fat synthesis and decomposition by initiating the AMPK signaling pathway. In addition, *L. plantarum* MTCC5690 and *L. fermentum* MTCC5689 can enhance the expression of fasting-induced adipocyte factor (FIAF), an inhibitor of lipoprotein lipase (LPL). LPL plays an important role in regulating lipid metabolism by preventing triglycerides from being stored in the form of fat [13]. Conversely, perturbations in the structure and activity of the gut microbiota propel the release of harmful metabolites, which impair the intestinal barrier, thereby triggering a constant change in the immune system and biochemical signals [13]. Specifically, the imbalance of the gut microbiota may increase the expression of lipopolysaccharide (LPS), which degrade the mucous layer. A “leaky” gut can facilitate the translocation of LPS from the intestine into the periphery, which is recognized by pattern recognition receptors, further activating several signal transduction pathways, such as NF-κB, MAPKs, PI3K/Akt, or producing pro-atherogenic trimethylamine (TMA), which ultimately causes metabolic disorders [14]. Therefore, interacting pathways such as the endocrine, metabolic, and immunological systems intertwine with other routes, providing bidirectional communication between the gut microbiota and host.

### 2.1. Altered Composition of Gut Microbiota

Research exhibited that patients with metabolic disorders have gut microbiome alterations compared with healthy controls, indicating that the gut microbial diversity receded and the pathogenic bacteria increased. Gut microbiota dysbiosis can impair gut permeability and then increase circulating LPS levels, which promote low-grade inflammation and, ultimately, metabolic disorders. The gut microbiota in animals with obesity displayed gut microbiological ecology disturbance, including the exasperation of the Firmicutes phylum, and the reduction of the Bacteroidetes phylum [15]. Epidemiological studies also revealed that the abundance of LPS-producing bacteria and LPS increased in various obese cohorts [16]. Fei et al. isolated *Enterobacter cloacae* B29 (an LPS-producing bacterium) from a morbidly obese human’s gut which was then transplanted to germ-free (GF) mice, which induced obesity and insulin resistance in GF mice [17]. In the population with T2D, the gut microbiota has been characterized by a decline in metabolically butyrate-producing microbiota and a compensatory expansion of the pathogenic bacteria that were known causes of various other diseases [6]. As an energy source for colonocytes, butyrate can effectively mitigate inflammation and oxidative stress, as well as protect gut barrier function [18]. In patients with steatosis, the abundance of *Lachnospiraceae* and *Ruminococcaceae* responsible for butyrate production were fewer, while the abundance of *Acidaminococcus*, *Escherichia* spp., and *Bacteroides* spp. related to insulin resistance was enriched [19].

### 2.2. Gut Microbiota-Derived Signaling Metabolites

Diet or body activities generate selection pressures that drive the gut microbiota to produce metabolites, in particular SCFAs, tryptophan metabolites, TMA, and so on. Through the production of these metabolites, the gut microbiota actively communicates with host cells, participating in the process of metabolic disorders. Meanwhile, these molecules also functionally interplay with other endocrine hormones, such as leptin, glucagon-like peptide 1 (GLP-1), and peptide YY [20]. Gut microbes employ dietary bioactive compounds to synthesize SCFAs (e.g., acetate, butyrate, propionate), which affect host metabolism by binding to G protein-coupled receptors. For instance, overweight adults supplemented with propionate stimulated the release of GLP-1, coupled with weight and liver steatosis reduction [21]. A butyrate precursor drug intervention in high-fat-fed mice improved diet-induced obesity and had a certain alleviating effect on hepatic steatosis and insulin resistance [22]. As distinct from butyrate and propionate, acetate may have a negative effect on obesity because it promotes hyperphagia by stimulating the secretion of ghrelin and contributes to fat storage by increasing the release of glucose-stimulated insulin [23]. Indole, a tryptophan microbial catabolite from *Bacteroides*, *Lactobacillus*, and *Bifidobacterium*, can improve intestinal barrier functions and enhance GLP-1 release, thereby indirectly effecting insulin secretion and appetite regulation [24,25]. Indole is metabolized to indole-3-propionic acid, which helps improve insulin secretion and sensitivity, reducing the incidence rate of T2D [26]. Besides, gut microbiota, primarily those from the families *Clostridia* and *Enterobacteriaceae*, can metabolize some dietary nutrients (e.g., lecithin, choline, and carnitine) as substrates to produce TMA [13]. And TMA is oxidized into trimethylamine-N-oxide (TMAO), participating in the processes of inflammation, cholesterol metabolism, and thrombosis. Research has revealed that TMAO increases the production of pro-inflammatory cytokines, decreases anti-inflammatory cytokines, and induces platelet hyperreactivity, thus facilitating atherosclerotic thrombotic events [27].

### 2.3. Fuelling Metabolic Inflammation

Chronic low-grade inflammation is another robust driver of metabolic syndromes. Dysbiosis or alteration in the composition of the gut microbiota composition and harmful metabolites contribute to the disruption of the intestinal barrier. Pioneering studies in animals and humans revealed that individuals with obesity, T2D, and NAFLD exhibited increased intestinal permeability [28,29,30]. Increased intestinal permeability allows hyper-translocation of harmful metabolites into the systemic circulation and metabolic organs, e.g., the liver and adipose tissue, thereby triggering metabolic inflammation [31]. Mechanically, microbial-associated molecular patterns, including LPS, can specifically be recognized by pattern recognition receptors (PRRs), such as toll-like receptors (TLRs) and NOD-like receptors (NLRs). PRRs activate several signal transduction pathways (e.g., NF-κB, MAPKs, and PI3K/Akt) via an adaptor molecule, MyD88, thus contributing to the breakdown of metabolic homeostasis [32]. Amar et al. found that a high-fat diet triggered the translocation of Escherichia coli through intestinal mucosa to mesenteric adipose tissue and increased the adherence in intestinal mucosa, fuelling a continuous metabolic bacteremia. Further analysis revealed that this phenotype was impeded in mice lacking Nod1, but overtly increased in Myd88 knockout and ob/ob mice [31]. It was worth noting that the probiotic strain Bifidobacterium animalis subsp. lactis 420 intervention for six weeks can reverse the bacterial translocation process, thereby improving inflammatory and metabolic status [32].

## 3. Gut Microbiota and Host Metabolism Variations during Fasting

Preclinical studies consistently demonstrated that the IF contributed to the reconstruction of the composition of gut microbes, with a major bloom in *Akkermansia muciniphila* and *Lactobacillus*, a reduction in pro-inflammatory taxa *Desulfovibrio* and *Turicibacter*, as well as enhancing antioxidative microbial metabolic pathways [33]. Interestingly, research showed that *A. muciniphila* could alleviate multiple energy dysmetabolism-induced diseases, whereas the lower abundance of this microbiota was positively correlated with hyperlipidemia, T2D, and fatty liver [4]. Fasting-induced adipocyte factor (FIAF) is an inhibitor of lipoprotein lipase (LPL) that is upregulated during fasting. Probiotics, such as *Lactobacillus plantarum* MTCC5690 and *Lactobacillus fermentum* MTCC5689, can also enhance the expression of FIAF. FIAF stimulates the oxidation of fatty acids, prevents triglycerides from being stored in the form of fat by inhibiting LPL, thereby regulating lipid metabolism, and protects against diet-induced obesity [13]. Gut rest (i.e., the IF) could also improve gut epithelial integrity and, as a result, mitigate the leakage of LPS and blunt systemic inflammation [34]. A study reported that a metabolic “switch” during fasting acts to promote energy balance by enhancing gut epithelial integrity [35]. In addition, the gut microbiota itself also follows diurnal oscillations in connectivity and robustness and is controlled by feeding regimes [36]. And the IF can coordinate the dynamic responses to modulate host health.

One mechanism is that properly fasting may coordinate the adaptive responses of the circadian rhythm. The central clock of the brain in the suprachiasmatic nucleus of the hypothalamus orchestrates circadian rhythm and subsequently coordinates the peripheral clock genes present in peripheral organs (e.g., the liver, heart, lungs, and kidneys) and immune cells [37]. A disrupted activity-rest cycle indirectly facilitates excessive caloric intake and perturbs the normal counter-regulatory metabolic state [38]. Nutrient-sensing pathways intimately interact with the circadian clock. Rodents are nocturnal animals that preferentially consume food preferentially during the dark phase. A high-fat diet propels the mice to eat around the clock, resulting in severely blunting the diurnal feeding rhythms and disturbing metabolic pathways entrained by both circadian and feeding rhythms [39]. Intriguingly, introducing the TRF effectively attenuates body weight gain and improves overt rhythms in mice with the high-fat diet [40]. Fasting regimens might enhance the robustness or amplitude of the circadian oscillation. Furthermore, the IF leads to liver glycogen store depletion and lipolysis of free fatty acids, which are metabolized to generate β-hydroxybutyrate (β-HB). Research showed that IF dietary regimes could develop a two-fold concentration of β-HB [33]. As a signaling mediator, this metabolite is involved in many regulations of cellular functions and adaptive responses, such as lipolysis, oxidative stress, and metabolic homeostasis [41]. IF seems to prevent weight gain and increase energy expenditure by triggering brown adipose tissue nonshivering thermogenesis as well as the browning of white adipose tissue [42]. Meanwhile, it also plays an essential role in metabolic disorders by alternating related-gene expression, activating cell surface receptors, and modifying histone [43].

The field of clinical endocrinology and metabolism has generated observational and efficacy data supporting a role for the IF in metabolic disorders by regulating gut microbiota. A randomized clinical trial from Guo et al. identified that the gut microbiota alteration attributed to the 8-week IF, coupled with distinct genetic shifts of carbohydrate metabolism, significantly attenuated the obesogenic effect, modulated inflammatory cytokines, and improved vasodilatory parameters [9]. In addition, in the genetic model of T2D mice, Wei et al. revealed that intervention with IF led to the alternation of gut microbiota, including increased abundance of *Parabacteroides* and *Blautia*, as well as reduced *Saccharbacteria*, *Prevotellaceae*, *Alistipes*, and *Ruminococcaceae*, which contributed to alleviating the deterioration of pancreatic islets and the loss of β cells [44].

## 4. Potential Role for Physical Exercise in the Modification of the Gut Microbiota in Metabolic Disorders

Physical exercise masters adaptational events in human metabolic equilibrium to enable extraordinarily beneficial actions. Physical exercise propels the motility of the bowel, changes the temperature and distribution of blood flow, and actively regulates the functioning of immune system cells in the gut mucosa, which helps maintain a healthy intestinal barrier by reducing chronic low-intensity inflammation and increasing the abundance of beneficial bacteria [45]. A substantial number of studies have tried to describe the effects of physical exercise on the gut microbiota composition of active versus non-active populations [46]. In active women, an elevation of a significant abundance of health-promoting bacteria such as *Faecalibacterium prausnitzii* and *A. muciniphila* was found [47]. Observational research conducted on humans and rodents all converged to indicate a reduction in the abundance of *Akkermansia* spp. In the gut of individuals with metabolic disorders such as obesity, T2D, NAFLD, and cardiovascular diseases (CVDs) [48,49,50].

### 4.1. Obesity

Regular and adequate levels of physical exercise favorably prevent weight gain and modify bacterial communities in mice with a high-fat diet and in sedentary adults with obesity. In general, physical exercise is beneficial to reducing visceral and subcutaneous adipose tissue volumes. The balance between Bacteroidetes and Firmicutes is directly related to obesity; levels are reduced in obese individuals. A study from Evans et al. revealed that intervention with voluntary wheel running for 12 weeks significantly increased the Bacteroidetes/Firmicutes ratio and the relative proportion of butyrate-producing bacteria in high-fat diet-induced obesity mice [51]. These results are in perfect concordance with population-based studies. Intervention with endurance exercise for six weeks increased butyrate-regulating bacterial taxa (*Lachnospira* spp., *Lachnospiraceae*, and *Faecalibacterium* spp.) and the fecal concentrations of acetate, butyrate, and propionate in sedentary adults with obesity [52]. Other animal studies supported that there was no change in the Bacteroidetes/Firmicutes ratio, but there was an increase in *Akkermansia* and a reduction in *Proteobacteria* after exercise intervention [53].

### 4.2. T2D

Many studies have reported that physical exercise promotes a wide cadre of favorable responses in reducing the incidence of T2D, together with obvious improvements in insulin sensitivity-related indexes and impaired glucose tolerance [45]. Remarkably, gut microbiota could play a leading role in influencing mechanisms of these processes. In patients with T2D, Motiani and colleagues revealed that print interval and moderate-intensity continuous exercise reduced systematic and intestinal inflammatory markers (tumor necrosis factor-α, LPS), increased the Bacteroidetes/Firmicutes ratio and decreased the abundance of *Clostridium* genus and *Blautia* [54]. Interestingly, a previous study revealed that *Blautia* was one of the most abundant genera in prediabetes and T2D compared with healthy subjects, which increased the release of pro-inflammatory cytokines [55,56]. In addition, different activity intensities also have different effects on the abundance and function of specific gut microbiota. Moderate intensity and prolonged exercise resulted in a decrease in *Clostridium* Cluster IV, *Bifidobacterium*, *A. municiphila*, and butyrate-producing taxa in more active people with T2D. Higher-intensity activity also increased butyrate producers, but from different orders (*Eryspelothrichales* and *Oscillospirales*) [57]. Butyrate may play an instrumental role in protecting the integrity of the gut barrier and assembling the expression of tight junctions. Amino acids, carbohydrates, cofactors, and vitamins, as well as amino acids and nucleotide sugar metabolic pathways, were also expressed differently between moderate intensity and higher intensity activity groups [57].

### 4.3. NAFLD

A characteristic of NAFLD is the accumulation of triglyceride in liver cells, which is formed from the esterification of fatty acids in the liver [58]. As an intricate disease, the pathophysiology of NAFLD is closely intertwined with insulin resistance, oxidative stress, inflammation responses, epigenetic modifiers, and others [59]. Insulin resistance in adipose tissue contributes to an incomplete suppression of lipase, promoting lipolysis and the release of FFA, which are elevated in serum and are taken up by the liver of NAFLD patients [60]. Intriguingly, it has been shown that physical exercise can improve liver status through hepatic or peripheral lipid metabolism, insulin sensitivity, and inflammation. The results of a randomized controlled trial for diabetic obese patients with NAFLDA revealed that high-intensity interval and moderate-intensity continuous exercise regulated lipid metabolism by reducing triglycerides and visceral lipids [61]. Physical exercise not only directly affects metabolic responses but also mediates its beneficial effects on NAFLD via regulating the gut microbiota. Several lines of evidence indicate that microbial populations are altered in patients with NAFLD compared with healthy controls. A study from Qin and colleagues revealed that NAFLD patients with advanced fibrosis were characterized by an exasperation of Proteobacteria and *Escherichia coli*, along with a decrease in Firmicutes [62]. Gut microbiota dysbiosis renders the bowel more permeable with a parallel release of LPS, which is transported over the intestinal lumen and reaches the liver via the portal vein, and initiates TLR signaling. In particular, the LPS-induced TLR4 cascade in hepatocytes triggers elevated systemic levels of proinflammatory cytokines, thus promoting insulin resistance, inflammation, and fibrosis [63]. Studies on exercise (a combined aerobic and resistance training protocol) in rodent models supported that exercise effectively promoted the enrichment of a functionally protective microbiota and increased the secretion of mucus in the intestinal mucus layer by upregulating the expression of intestinal tight-junction proteins, which, in turn, prevented disturbance of the gut-liver axis and controlled hepatic lipid metabolism [64].

### 4.4. CVDs

The gut microbiota participates in the pathogenesis of CVD through regulating the production of TMA and LPS, triggering bacterial translocation to carotid arterial plaques, and increasing blood pressure [65,66]. Noteworthy, physical exercise can increase the flow-mediated shear stress on the artery walls, decrease serum triglyceride levels, as well as enhance cardiac reserve capacity and autonomic regulation, which contribute to improving endothelial function and reducing the prevalence of coronary heart diseases and cardiomyopathies [67,68]. As a unique form of physiological stress, regular physical exercise can improve intestinal peristalsis. This “internal activity” may boost the shedding of loosely bound microbes in the intestinal epithelium, thereby promoting the growth of healthy commensals that are involved in the development of healthy mucosal immunity [69]. Compared to mice that received microbiota transplants from non-exercised mice, mice that received microbiota from exercised mice exhibited better cardiac function, as indicated by recent studies. Mechanistically, physical exercise alternates gut microbial richness and community structure as well as propels the release of 3-hydroxyphenylacetic acid and 4-hydroxybenzoic acid, protecting against cardiac dysfunction in myocardial infarction mice [70].

## 5. Modulation Effects of Combining IF and Physical Exercise on Metabolic Disorders

**Figure 2:**
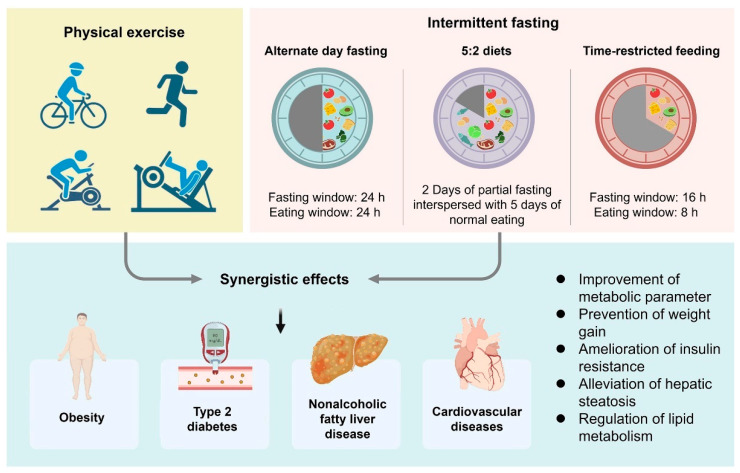
Combining physical exercise and IF for preventing metabolic disorders.

As discussed above, IF and physical exercise are simple options implementable in daily life situations to prevent metabolic disorders. Noteworthy, ample experimental evidence has indicated that the combination of exercise and IF lead to a greater improvement of metabolic parameters (Figure 2 and Table 1). The interaction between IF and exercise may have potentiated the hormonal effects of insulin metabolism and is associated with higher glycemic tolerance, which is attributed to the increase in AMPK activity and GLUT4 protein [71]. In a 3-month randomized parallel-arm trial, the results demonstrated that ADF combined with aerobic exercise was effective for reducing intrahepatic triglycerides in adults with obesity and NAFLD [72]. Combining IF and physical exercise produces superior changes in manipulating lipid levels. Bhutani et al. assessed the effects of a 12-week combination of ADF and exercise (3 d/week) on lipid levels in obese adults. Results revealed a significant reduction in low-density lipoprotein and an increase in high-density lipoprotein concentrations in the combination group only [73]. A comparative and randomized cross-over experiment revealed that high-intensity interval exercise in the fasted state led to a significant reduction in fat mass in adult women compared to exercise intervention alone [74]. Batitucci et al., in a randomized study, found that the 5:2 IF protocol with high-intensity interval exercise efficiently promoted increments in fat-free mass compared to exercise in isolation and improved the functional physical capacities in women with obesity [75]. Furthermore, in diet-induced obese mice, IF with high-intensity interval exercise resulted in significantly preventing weight gain in the form of fat mass accumulation and reducing serum low-density lipoproteins levels compared to not combining exercise [76].

The synergistic mechanisms of IF and physical exercise are still poorly explored, especially in the gut microbiota. On the one hand, combined IF and exercise can drastically change the ways our bodies synthesize and utilize fuel sources. When exercising in a fed state, the body primarily utilizes glucose from the recent diet as the predominant source of fuel. However, when exercising in a fasted state, as a result of the depletion of glucose and glycogen stores, the preferential fuel comes from lipolysis and fat oxidation, particularly the breakdown of intramyocellular triglycerides, resulting in stronger beneficial actions for metabolic health [77]. Some studies revealed that the combination of IF and exercise increased mitochondrial activity, exacerbated the consumption of stored lipids, and enhanced the lipid reserves in muscle [78]. And the reservation of lipid in muscle is an important substrate source during acute exercise, which is considered to be a beneficial adaptation to exercise in the fasting state [79]. It is still unclear whether a combination of IF and exercise can affect the gut microbiota towards a healthy state. We found through searching the National Center for Biotechnology Information database that only an animal experiment evaluated the effects of the combination of exercise and IF on the microbiome. Unexpectedly, the combination group showed a lower abundance of *Bifidobacterium* and *Lactobacillus* compared to the other groups but displayed a tendency towards an anxiolytic effect [80]. Therefore, more animal and human studies about the effects of the combination of exercise and IF on the microbiome merit further investigation to determine the regulation mechanisms.

## 6. Conclusions

Presently, health concepts such as exercise, fitness, and a balanced diet have become a consensus, which also guides us to actively pursue a healthy lifestyle. IF and physical exercise promote adaptational changes in human metabolic capacities to enable extraordinarily beneficial actions. But the receptiveness of an individual to these two intervention methods varies significantly. There is great diversity in the patterns of IF and modalities of exercise. The research on how to combine IF and exercise is contradictory. It must be highlighted that fasting and exercise cannot always be carried out simultaneously, as both are sources of stress. Therefore, balancing the timing of exercise and fasting, such as which intensity of exercise or no exercise to engage in during a fasted state, is of fundamental importance. Age differences among individuals are also an important factor to consider during a combined IF and exercise intervention. In addition, a few ongoing studies are currently investigating the effects of combined exercise and fasting on the gut microbiota. Deciphering the pathogenesis of these processes represents a key challenge in preventing metabolic disorders.

## Figures and Tables

**Table 1:** Effects of physical exercise and IF on metabolic disorders.

Subjects	Methodological Approach	Duration of Intervention	Affected Gut Microbiota	Experimental Results	Reference
Adults with metabolic syndrome	Randomized clinical trial	“2-day” modified IF for 8 weeks	Ruminococcaceae ↑ Roseburia ↑	Reduce fat mass; Ameliorate oxidative stress; Modulate inflammatory cytokines; Improve vasodilatory parameters	[9]
			Increase the production of SCFAs;		
			Decrease the circulating levels of LPS;		
Mice with T2D	Animal experiments	IF for 8 weeks	Parabacteroides ↑	Improve insulin sensitivity and β cell function Reduce hepatic steatosis	[44]
			Blautia ↑		
			Prevotellaceae ↓		
			Alistipes ↓		
			Ruminococcaceae ↓		
High-fat diet -induced obesity mice	Animal experiments	Voluntary wheel running for 12 weeks	Increase the Bacteroidetes/Firmicutes ratio and the relative proportion of butyrate-producing bacteria	Prevent body weight gain and adiposity	[51]
Lean and obese adults	Human experiments	Endurance exercise for 6 weeks	Increase butyrate-regulating bacterial taxa (*Faecalibacterium* spp., *Roseburia* spp., *Lachnospira* spp., *Lachnospiraceae*, and *Clostridiales* spp.)	Decrease body fat percentage;	[52]
				Increase bone mineral density;	
				Improve cardiorespiratory fitness	
People with T2D	Randomized clinical trial	Combined aerobic and resistance moderate intensity exercise, or combined aerobic and resistance high-intensity exercise for 8-weeks	Increase *Bifidobacterium*, *Akkermansia municiphila*, and butyrate-producing taxa	-	[57]
Patients with NAFLD	Randomized clinical trial	Alternate-day fasting combined with exercise for 3 months	-	Reduce hepatic steatosis;	[72]
Active women (27 ± 6 years)	Comparative and randomized cross-over trial	High-intensity interval training combined with IF for 16 weeks	-	Decrease in fat mass;	[74]
				Increase in jumping performance	
Women with obesity (32.2 ± 4.4 years)	Randomized clinical trial	5:2 IF protocol with high-intensity interval exercise for 8 weeks	-	Promote increments in fat-free mass;	[75]
				Improve physical fitness and strength	

↑ = up-regulate, ↓ = down-regulate.
